# An evaluation of the InDevR FluChip-8G insight microarray assay in characterizing influenza a viruses

**DOI:** 10.1186/s40794-021-00133-7

**Published:** 2021-03-17

**Authors:** Emily S. Bailey, Xinye Wang, Mai-juan Ma, Guo-lin Wang, Gregory C. Gray

**Affiliations:** 1 Division of Infectious Diseases, Duke University School of Medicine, Durham, North Carolina USA; 2 Duke Global Health Institute, Duke University, Durham, North Carolina USA; 3 Julia Jones Matthews Department of Public Health, Texas Tech University Health Sciences Center, Abilene, TX USA; 4 Global Health Research Center, Duke Kunshan University, Kunshan, China; 5 School of Medical Sciences, Faculty of Medicine, University of New South Wales, Sydney, NSW Australia; 6 State Key Laboratory of Pathogen and Biosecurity, Beijing Institute of Microbiology and Epidemiology, Beijing, China; 7 Emerging Infectious Diseases Program, Duke-NUS Medical School, Singapore, Singapore

**Keywords:** Clinical performance, Diagnostic validation, FluChip-8G insight assay, Influenza subtyping, Microarray, Multiplex RT-PCR

## Abstract

Influenza viruses are an important cause of disease in both humans and animals, and their detection and characterization can take weeks. In this study, we sought to compare classical virology techniques with a new rapid microarray method for the detection and characterization of a very diverse, panel of animal, environmental, and human clinical or field specimens that were molecularly positive for influenza A alone (*n* = 111), influenza B alone (*n* = 3), both viruses (*n* = 13), or influenza negative (*n* = 2) viruses. All influenza virus positive samples in this study were first subtyped by traditional laboratory methods, and later evaluated using the FluChip-8G Insight Assay (InDevR Inc. Boulder, CO) in laboratories at Duke University (USA) or at Duke Kunshan University (China). The FluChip-8G Insight multiplexed assay agreed with classical virologic techniques 59 (54.1%) of 109 influenza A-positive, 3 (100%) of the 3 influenza B-positive, 0 (0%) of 10 both influenza A- and B-positive samples, 75% of 24 environmental samples including those positive for H1, H3, H7, H9, N1, and N9 strains, and 80% of 22 avian influenza samples. It had difficulty with avian N6 types and swine H3 and N2 influenza specimens. The FluChip-8G Insight assay performed well with most human, environmental, and animal samples, but had some difficulty with samples containing multiple viral strains and with specific animal influenza strains. As classical virology methods are often iterative and can take weeks, the FluChip-8G Insight Assay rapid results (time range 8 to 12 h) offers considerable time savings. As the FluChip-8G analysis algorithm is expected to improve over time with addition of new subtypes and sample matrices, the FluChip-8G Insight Assay has considerable promise for rapid characterization of novel influenza viruses affecting humans or animals.

## Introduction

Infectious diseases threatening human and animal populations are increasingly emerging from zoonotic sources. In regions, such as Southeast Asia, where there is intense and frequent contact between humans and animals, viral transmission between species can be rapid and quickly cause epidemics [1]. Of particular concern are viruses such as avian influenza viruses, swine influenza viruses, and zoonotic coronaviruses, which can cause severe morbidity and mortality [2, 3]. In particular, avian H5 and recently H7 influenza A strains have been problematic.

Our multinational team has previously collected field samples for influenza viruses in both the United States [4, 5] and in China [6, 7]. Our goal in this study was to examine a panel of animal, environmental, and human field samples collected in both the United States and China with evidence of influenza A or B virus against both classical virologic techniques and a new microarray assay, the FluChip-8G (FC8G) Insight Assay (InDevR Inc. Boulder, CO) at our laboratories at Duke University in Durham, NC and at Duke Kunshan University (DKU) in Kunshan China.

## Materials and methods

We sought to compare traditional laboratory detection and subtyping methods (partial or full genome sequencing, or real time PCR subtyping) with the new FluChip-8G Insight Assay technology. Published real-time polymerase chain reaction (qPCR) and real-time reverse transcription polymerase chain reaction (qRT-PCR) assays for influenza A and B viruses were used with cDNA positive controls and nuclease-free water as negative controls.

### Sample processing

For bioaerosol samples and clinical laboratory specimens, viral RNA was extracted from each 200 μl sample and eluted in 50 μl of elution buffer using a QIAamp MinElute Virus Spin Kit (Cat. No. 57704, Qiagen Inc., Hilden, Germany). Extracted RNA was then tested for the presence/absence of influenza A [8] and influenza B [9] by real-time reverse transcriptase polymerase chain reaction (qRT-PCR) assays using the SuperScript III Platinum One-Step qRT-PCR Kit (Thermo Fisher Scientific Inc., Waltham, MA). Each qRT-PCR run had positive and negative template controls. qRT-PCR reactions were performed in a 20 μL reaction mixture containing 5 μL template RNA under the following conditions: 50 °C for 30 min, 95 °C for 2 min, 45 cycles of 95 °C for 15 s, and 55 °C for 30 s.

For pig oral secretion and environmental samples, viral RNA was extracted using a MiniBEST viral RNA/DNA Extraction Kit (Cat. No. 9766, TaKaRa, Dalina, China). Extracted RNA was screened for influenza A by a qRT-PCR assay targeting the influenza matrix genome segment [10] using a One-Step RT-PCR kit (Cat. No. 56046, TaKaRa, Dalian, China). Each qRT-PCR run had positive and negative template controls. qRT-PCR reactions were performed under the following conditions: 42 °C for 30 min, 94 °C for 2 min, 40 cycles of 94 °C for 15 s, and 55 °C for 30 s.

All samples with quantification cycles (Cq) values ≤38 were considered positive for the influenza A virus matrix gene. But only positive samples with Ct values ≤30 were then inoculated onto MDCK cells or into 9–11 day old embryonated chicken eggs in an attempt at virus isolation. Virus isolation procedures were carried out in accordance with relevant institutional guidelines.

### Conventional PCR sequencing

For cultured samples, the entire viral genome was then amplified using a pair of universal primers [11] and a One-Step Conventional PCR kit (Cat. No. 055A, TaKaRa, Dalian, China). The amplified DNA was then purified using a MinElute PCR Purification Kit (Cat. No. 28004, QIAGEN) and sequenced on an Ion Torrent Personal Genome Machine (PGM, Life Technologies, San Francisco, CA, USA). Phylogenetic analysis was performed using MEGA 6 (University of Pittsburgh, Pittsburgh, PA). The maximum likelihood method was used in this analysis.

For remaining samples which may have the potential for multiple viruses or PCR inhibitors, RT-PCR was performed on the extracts using the hemagglutinin (HA) and neuraminidase (NA) gene primers described in Hoffmann et al., 2011 [12]. Invitrogen SuperScript III Reverse Transcriptase kit (Cat# 18080044, ThermoFisher Scientific Inc., Carlsbad, CA, USA) was used to convert RNA to cDNA and the FastStart High-fidelity PCR System (Roche Molecular Systems, Inc., Pleasanton, CA, USA) was used to amplify cDNA. Both kits were used according to the manufacturer’s instructions. Sequencing of resultant amplified product from both the HA and NA assays was performed by Eton Bioscience (Eton Bioscience, Inc., Raleigh, NC, USA) or by Baygene (Baygene, Beijing, China). Sequences were then aligned, edited, and compared to the NCBI sequence database using the BLAST application of BioEdit 7.1.9 (Ibis Biosciences, Carlsband, CA, USA). If samples could not be identified through conventional PCR and partial genome sequencing, follow up real time PCR subtyping was performed [8].

### FluChip-8G insight assay

The FluChip-8G Insight Assay (InDevR Inc., Boulder, CO) is a multiplexed RT-PCR and microarray-based assay. The focus of this assay is to detect nucleic acid from both seasonal and non-seasonal influenza A and B viruses by using a universal priming mixture to amplify full length HA, NA, M, NS, and NP gene segments of these viruses, followed by hybridization and detection using a DNA microarray. A pattern recognition-based approach is used to characterize the virus or viruses present. This technique is further described in recent studies [13, 14]. The FluChip-8G Insight Assay was performed according to manufacturer’s instructions. The FluChip-8G Insight Assay is based on two tiers of artificial neural networks in its pattern-based identification of influenza. In the first tier, influenza A, H1N1 pandemic 2009, and seasonal H3N2 influenza are differentiated from non-seasonal influenza. Tier 2 neural networks further identify non-seasonal influenza A subtypes including H1, H3, H5, H7, H9, N1, N2, N7, N8, and N9.

## Results

A total of 122 influenza A-, influenza B-, or influenza A- and B-positive samples were subtyped using standard subtyping methods including partial or full genome sequencing methods, or real-time PCR subtyping (Table 1). A breakdown of the number of samples tested by subtype and host species is provided in Table 1.

**Table 1:** The breakdown of the number of samples tested by subtype and host species

	human	avian	swine	Environment (bioaerosols/water)	Total
Influenza A					
Influenza A/H1N1pdm2009	22	–	3	4	29
Influenza A/seasonal H3N2	4	–	–	–	4
Influenza A/Non-Seasonal					76
H1N1	–	–	18	1	–
H1N2	2	–	–	–	–
H3N2	–	–	16	–	–
H3N8	–	1	–	–	–
H5N1	–	–	–	2	–
H5N6	–	3	–	1	–
H5N8	–	1	–	–	–
H7N9	–	7	–	5	–
H9N2	–	10	–	9	–
Influenza B	3	–	–	–	3
Dual Infection	10	–	–	–	10
Negative	2	–	–	2	4

All samples were also evaluated using the FluChip-8G Insight Assay system and classical virologic techniques. Individually, 59 (54.1%) of 109 influenza A-positive, 3 (100%) of the 3 influenza B-positive, and 0 (0%) of the 10 both influenza A- and B-positive samples were correctly identified by the FluChip-8G Insight Assay when compared to the classical techniques (Table 2). Despite this, it is important to note that in these mixed samples, one virus was identified correctly in all samples. Among the non-seasonal influenza-A samples, environmental samples were most commonly identified correctly (Table 3), with over 75% of samples identified correctly for H1, H3, H7, H9, N1, and N9. Similarly, for avian samples, over 126 samples were identified correctly with the exception of the N6 subtype. In contrast, there was low agreement with the swine samples for nearly all subtypes.

**Table 2:** The agreement of the “clinical”/first tier FluChip results to subtype determined by real-time PCR and sequencing

	Correctly Identified by FluChip	Not Identified by FluChip	% Correct
Influenza A			
Influenza A/H1N1pdm2009	26	3	90%
Influenza A/seasonal H3N2	4	–	100%
Influenza A/Non-Seasonal	29	47	38%
Influenza B	3	–	100%
Dual Infection	–	10	0%
Negative	3	1	75%

**Table 3:** The agreement of the Non-seasonal Subtyping FluChip results to subtype determined by real-time PCR and sequencing

	# correctly identified/total analyzed (%)			
	Swine samples	Avian samples	Environmental samples	Total
Non-seasonal HA subtyping				
H1	11%	–	100%	16%
H3	25%	100%	–	29%
H5	–	100%	100%	100%
H7	–	71%	80%	75%
H9	–	90%	78%	84%
Non-seasonal NA Subtyping				
N1	56%	–	100%	62%
N2	19%	90%	22%	40%
N6	–	33%	0%	25%
N8	–	100%	–	100%
N9	–	57%	80%	67%

## Discussion

In this field validation study, we conducted standard molecular detection analysis alongside a new technology for detecting and subtyping influenza A and B. Our overall goal was to determine if FluChip-8G Insight Assay technology was comparable to standard subtyping techniques, understanding that the technology was to date untested on animal-origin samples in challenging environmental and aerosol matrices. Our unique approach of challenging this new diagnostic tool with human, animal, and environmental samples is based on the One Health approach, which brings together researchers across disciplines for the improvement of human, animal, and environmental health. The research presented here demonstrates that subtyping influenza can vary across various sample types and that, although much research has been done on the human side of influenza transmission, important and novel variants are emerging at the human animal interface.

In this validation study, we examined a diverse panel of influenza A positive specimens. The FluChip-8G Insight Assay did not perform as well as we had hoped. Perhaps this is not surprising as many of the discrepant samples were swine oral secretion samples, which are complex matrices of dirt and oral fluid from pigs that have been previously described as difficult to subtype [15–17] and on which the assay analysis algorithm was not trained or optimized. Additionally, the viral RNA in specimens was not quantified and the multiplexed FluChip-8G Insight Assay may require more RNA than singleplex molecular methods. Another important discrepancy occurred when both influenza A and B (dual infection) was identified in human clinical samples by real-time PCR subtyping but only influenza A was detected by the FluChip-8G Insight Assay. As this diagnostic tool is designed to pick up both types of influenza, this is important to note; however, for clinical diagnostics, it may be that the detection of influenza is the most important result.

Much of the work presented here is supported by several previous studies evaluating the FluChip-8G Insight Assay technology [13, 14, 18]. First, two of the most commonly misidentified subtypes in are swine H3N2 and swine H1N1. This is similar to the results presented in Taylor et al., 2019, who suggested that genetic similarity of swine H3N2 and swine H1N1 viruses to the seasonal H1N1pdm09 virus (shared some specific gene segments, such as M, NS, NP, and HA) resulted in the misidentification [13]. Hence, when the viral concentration in samples with these specific subtypes is low, these samples may be misidentified. In order to obtain more accurate results in samples with these two particular subtypes, it may be necessary to determine the concentration of viral RNA and only accept a specific threshold of RNA prior to running the FluChip-8G Insight Assay system. Second, some samples with dual infections were only detected as one subtype by the FluChip-8G Insight Assay. This finding might be explained by the Blair et al. (2019) study [14]. Blair et al., 2019 suggested that if two subtypes co-exist in one sample, the subtype with the lower concentration may not be successfully detected. Future studies examining both samples with similar genetic subtypes and samples with dual infections are needed.

To our knowledge, this is the first challenge of the FluChip-8G Insight Assay with bioaerosol and environmental samples containing animal-origin influenza viruses and represented an interesting challenge. This research was largely exploratory and it included quite a difficult challenge for this new technology. We examined a convenience sample of assorted field specimens positive for diverse animal influenza viruses. Some of the specimens’ concentration of influenza RNA were likely relatively low. Even so, the FluChip-8G Insight Assay performed fairly well. As the FluChip-8G Insight Assay’s software is “trained” with data from samples such as those described herein and improved over time, we can expect the diagnostic results to improve. Certainly, the FluChip-8G Insight Assay holds much promise as a valuable diagnostic tool in that it greatly reduces the diagnostic time to identify and characterize novel influenza A and B viruses as compared to standard influenza molecular subtyping and sequencing methods which may take weeks.

## Data Availability

The data collected during the current study are available from the corresponding author on reasonable request.

## References

[1] Woolhouse ME, Gowtage-Sequeria S (2005). Host range and emerging and reemerging pathogens. Emerg Infect Dis.

[2] Coker R, Rushton J, Mounier-Jack S, Karimuribo E, Lutumba P, Kambarage D, Pfeiffer DU, Stärk K, Rweyemamu M (2011). Towards a conceptual framework to support one-health research for policy on emerging zoonoses. Lancet Infect Dis.

[3] Coker RJ, Hunter BM, Rudge JW, Liverani M, Hanvoravongchai P (2011). Emerging infectious diseases in Southeast Asia: regional challenges to control. Lancet.

[4] Borkenhagen LK, Wang GL, Simmons RA, Bi ZQ, Lu B, Wang XJ, Wang CX, Chen SH, Song SX, Li M, Zhao T, Wu MN, Park LP, Cao WC, Ma MJ, Gray GC. High Risk of Influenza Virus Infection Among Swine Workers: Examining a Dynamic Cohort in China. Clin Infect Dis. 2020;71(3):622-9. 10.1093/cid/ciz865. 10.1093/cid/ciz865 PMC7108185 31504322

[5] Anderson BD (2018). Prospective surveillance for influenza. virus in Chinese swine farms. Emerg Microbes Infect.

[6] Ma M, Anderson BD, Wang T, Chen Y, Zhang D, Gray GC, Lu J (2015). Serological evidence and risk factors for swine influenza infections among Chinese swine Workers in Guangdong Province. PLoS One.

[7] Ma MJ, Wang GL, Anderson BD, Bi ZQ, Lu B, Wang XJ, Wang CX, Chen SH, Qian YH, Song SX, Li M, Lednicky JA, Zhao T, Wu MN, Cao WC, Gray GC (2018). Evidence for cross-species influenza a virus transmission within swine farms, China: a one health, Prospective Cohort Study. Clin Infect Dis.

[8] WHO. WHO information for molecular diagnosis of influenza virus. Available from: http://www.who.int/influenza/gisrs_laboratory/molecular_diagnosis/en. Accessed 8 June 2019.

[9] Selvaraju SB, Selvarangan R (2010). Evaluation of three influenza a and B real-time reverse transcription-PCR assays and a new 2009 H1N1 assay for detection of influenza viruses. J Clin Microbiol.

[10] Fouchier RA (2000). Detection of influenza a viruses from different species by PCR amplification of conserved sequences in the matrix gene. J Clin Microbiol.

[11] Zhou B, Donnelly ME, Scholes DT, St. George K, Hatta M, Kawaoka Y, Wentworth DE (2009). Single-reaction genomic amplification accelerates sequencing and vaccine production for classical and swine origin human influenza a viruses. J Virol.

[12] Hoffmann E, Stech J, Guan Y, Webster RG, Perez DR (2001). Universal primer set for the full-length amplification of all influenza a viruses. Arch Virol.

[13] Taylor AW, Dawson ED, Blair RH, Johnson JE, Slinskey AH, Smolak AW, Toth E, Liikanen K, Stoughton RS, Smith C, Talbot S, Rowlen KL (2019). Analytical evaluation of the microarray-based FluChip-8G influenza a+B assay. J Virol Methods.

[14] Blair RH, Dawson ED, Taylor AW, Johnson JE, Slinskey AH, O’Neil K, Smolak AW, Toth E, Liikanen K, Stoughton RS, Smith CB, Talbot S, Rowlen KL (2019). Clinical validation of the FluChip-8G influenza a+B assay for influenza type and subtype identification. J Clin Virol.

[15] Decorte I, Steensels M, Lambrecht B, Cay AB, de Regge N (2015). Detection and isolation of swine influenza a virus in spiked Oral fluid and samples from individually housed, experimentally infected pigs: potential role of porcine Oral fluid in active influenza a virus surveillance in swine. PLoS One.

[16] Janke BH (2014). Influenza a virus infections in swine: pathogenesis and diagnosis. Vet Pathol.

[17] Romagosa A, Gramer M, Joo HS, Torremorell M (2012). Sensitivity of oral fluids for detecting influenza a virus in populations of vaccinated and non-vaccinated pigs. Influenza Other Respir Viruses.

[18] Townsend MB, Dawson ED, Mehlmann M, Smagala JA, Dankbar DM, Moore CL, Smith CB, Cox NJ, Kuchta RD, Rowlen KL (2006). Experimental evaluation of the FluChip diagnostic microarray for influenza virus surveillance. J Clin Microbiol.

